# What are the tools provided by the EU to support health and care improvement?

**DOI:** 10.1093/eurpub/ckac129.010

**Published:** 2022-10-25

**Authors:** N Mauer

**Affiliations:** European Observatory on Health Systems and Policies, Brussels, Belgium

## Abstract

Improving health and care systems is primarily the responsibility of Member States within the EU. Nevertheless, the EU provides a lot of potential support to Member States that can help to improve health systems. However, the range of EU tools that can help to strengthen health systems has grown up over decades across different policy fields, and most potential EU support is incorporated within instruments that have wider objectives. This can make it challenging to identify and optimise the use of the different instruments, as well as to create synergies across tools as part of an overall process of improving a health system. For example, the EU's wide-ranging support across diverse instruments and policy areas has contributed towards improving the prevention, monitoring, and management of cancer, as well as modernising dedicated health infrastructure and developing common cancer policy guidance in Europe over several decades. This presentation will help with understanding what different tools can be used for, showcasing content from the policy brief and how it can be helpful with addressing the challenges faced by health policy makers. Using concrete case studies, it will illustrate how different instruments might be combined in pursuit of a specific process of health system strengthening and transformation.

